# Advances in Targeted Microbeam Irradiation Methods for Live *Caenorhabditis elegans*

**DOI:** 10.3390/biology13110864

**Published:** 2024-10-24

**Authors:** Michiyo Suzuki

**Affiliations:** Department of Quantum-Applied Biosciences, Takasaki Institute for Advanced Quantum Science (TIAQS), National Institutes for Quantum Science and Technology (QST), 1233 Watanuki, Takasaki 370-1292, Gunma, Japan; suzuki.michiyo@qst.go.jp; Tel.: +81-(0)27-346-9542; Fax: +81-(0)27-346-9353

**Keywords:** microbeam, heavy ion, proton, *Caenorhabditis elegans*, immobilization, anesthesia-free, microfluidic chip

## Abstract

Charged-particle microbeam irradiation devices, which can convert heavy-ion or proton beams into microbeams and irradiate individual animal cells and tissues, have been developed and used for bioirradiation in Japan, the United States, China, and France. Microbeam irradiation technology has been used to analyze the effects of irradiation on mammalian cancer cells, especially bystander effects. In 2006, individual-level microbeam irradiation of the nematode *Caenorhabditis elegans* was first realized using JAEA-Takasaki’s (now QST-TIAQS’s) TIARA collimated microbeam irradiation device. As of 2023, microbeam irradiation of *C. elegans* has been achieved at five sites worldwide (one in Japan, one in the United States, one in China, and two in France). This paper summarizes the global progress in the field of microbeam biology using *C. elegans*, while focusing on issues unique to microbeam irradiation of live *C. elegans*, such as the method of immobilizing *C. elegans* for microbeam experiments.

## Simple Summary

The nematode *Caenorhabditis elegans*, which is only 1 mm long, is used as a model to study the effects of irradiation on tissues (organs) of living organisms. This paper reviews the development of irradiation techniques using charged-particle microbeams in Japan, the U.S., China, and France, in which heavy-ion and proton beams (types of ionizing radiation) are targeted to specific cells or regions of *C. elegans*, and outlines the progress made over the past 20 years and where we are today. An essential part of irradiating the targeted cell/tissue of microscopic animals is having an immobilization method which does not damage the animals’ physiological activity. This article introduces some technical difficulties that differ from those concerning the irradiation of cultured cells, which have been the main target of irradiation in the past.

## 1. Introduction

The elaborate and complex biological functions of individual animals (e.g., sensory responses, higher neural functions such as learning and memory, motor generation and control, developmental control, immune responses, anti-aging) are achieved by the cooperation of many cells with different functions. Understanding complex biological functions requires a comprehensive analysis that broadens the perspective to the cellular network, that is, at the cell, tissue, and individual levels, including not only the role of genes but also the physical connections between individual cells [1].

Microbeam irradiation of charged particles such as heavy ions or protons has attracted attention as a technique for targeting a certain cell and/or tissue. Charged particles such as heavy ions or protons have characteristic biological effects such as high cell killing and characteristic mutagenesis, and have been applied in cancer therapy and ion-beam-breeding [2]. In the conventional broad-beam irradiation method used to study the effects of ionizing radiation on cell culture response and ion-beam breeding, samples are irradiated whole, but it has been difficult to elucidate the cellular response induced by a single ion hit. As a way to overcome this problem, microbeam irradiation has been considered as a method to target and irradiate a specific region of a biological sample under a microscope with a micrometer-sized ion-beam spot, which is smaller than a cell [2].

Microbeam irradiation can deliver a type of “external stimulus” in the form of ionizing radiation to specific cells and tissues at very precisely controlled doses. It is therefore possible not only to analyze the dose- and site-dependent effects of radiation, but also to derive the conditions under which cells and tissues are temporarily inactivated or enhanced. As this can be done by controlling the dose and irradiation site without permanently destroying the cells and tissues, it provides an effective tool for estimating their roles in various biological functions [1,2].

The development of technology for both life science research using microbeams and heavy-ion/proton microbeam irradiation of tissues and individuals has been intensively conducted at two sites in Japan, one site in the USA, one site in China, and two sites in France. Studies using microbeam irradiation can be divided into three main categories: studies of ion-hit effects on cells, studies of radiation-induced bystander effects, and studies to elucidate the functions of individual organisms through local heavy-ion irradiation. In the 2000s, irradiation of cultured cells was the mainstay for analyzing bystander responses at the molecular and cellular levels, but recently there has been growing momentum worldwide to extend the scope of this research to the tissue and individual levels [1,2,3,4,5,6,7].

Research is also beginning to explore the roles of individual cells and tissues in supporting the precise biological functions of individual animals, rather than simply analyzing the effects of ionizing radiation. Specifically, targeted stimulation of specific cells in individual animals causes changes in cellular activity and can help to investigate how these cells are involved in the realization of sophisticated biological functions, such as the control of whole-body movement and the development of the central nervous system. A technique of targeting and irradiating a local area of an individual nematode, which is considerably thicker than a cell, with different irradiation levels has been established and is opening the door to a new research paradigm of analyzing the mechanisms of various biological functions using heavy-ion microbeams [1].

**Figure 1:**
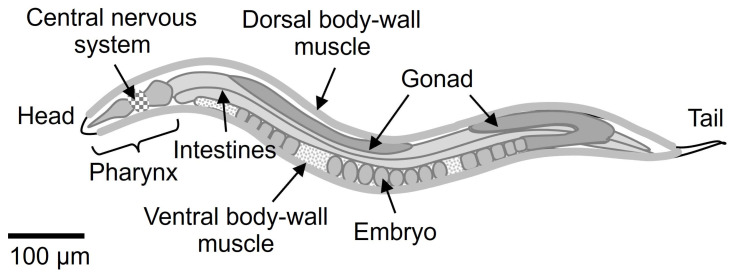
Schematic of the nematode *Caenorhabditis elegans* hermaphrodite.

The nematode *Caenorhabditis elegans* hermaphrodite consists of only 959 cells and contains a neural information processing system, a muscle motor control system, and digestive and reproductive organs (see Figure 1). It is the only multicellular organism in which the arrangement of all cells and intercellular connections is completely understood. It is an attractive model organism for understanding the operating principles of diverse biological functions such as individual development and aging, higher neural functions such as learning and memory, behavioral control such as stimulus response, digestion, metabolism, and reproduction. The effects of ionizing radiation on *C. elegans* have been studied on behaviors such as chemotaxis, associative learning, and motor control, in addition to germ cell response and aging, providing important insights into the biological effects of acute/chronic broad-beam irradiation [8,9]. Based on this knowledge, *C. elegans* has been chosen as the starting point for microbeam irradiation of individual living organisms at five microbeam irradiation facilities worldwide [10,11,12,13,14,15,16,17,18,19,20,21,22].

This paper summarizes recent advances in the analysis of biological functions using microbeam irradiation, with live *C. elegans* as the target organism.

## 2. Recent Advances in Microbeam Irradiation of Individual Animals

There are two sites in Japan with microbeam facilities capable of irradiating individual animals. The first is the Takasaki Ion Accelerators for Advanced Radiation Application (TIARA) at the Takasaki Advanced Radiation Research Institute of the Japan Atomic Energy Agency (JAEA-Takasaki; now Takasaki Institute for Advanced Quantum Science (TIAQS), part of the National Institutes for Quantum Science and Technology (QST)). At this site, two heavy-ion microbeam irradiation devices for biological sample irradiation, each using a different method of microbeam formation, are installed on the vertical beamline of the AVF cyclotron. The collimated heavy-ion microbeam irradiation device installed at the HZ1 port has been used for microbeam irradiation of biological samples since 1994 [1,2,3,4,6]. The focused heavy-ion microbeam irradiator installed at the HX1 port has been under development since 2005 for more precise cell irradiation [4]. In both facilities, a wide range of ion beams accelerated by the AVF cyclotron are progressively miniaturized and extracted into the atmosphere, allowing the irradiation of biological samples on a stage to be observed under a microscope as the ions are delivered to their target sites. At TIARA, the main targets are the nematode *C. elegans* [10,11,12,13,14,15], medaka (*Oryzias latipes*) [23,24], and silkworm (*Bombyx mori*) [25,26].

The other site, also at QST, is the Institute for Quantum Medical Science, where proton beams from an electrostatic accelerator can be narrowed down to less than 5 µm by a microbeam facility, SPICE (Single Particle Irradiation system to Cell) [27]. In terms of irradiating individual animals, proton microbeam irradiation of zebrafish embryos has been achieved [28,29].

Proton microbeam irradiation of *C. elegans* individuals has been carried out in the USA at the Radiological Research Accelerator Facility of Columbia University (RARAF) [16,17] and in China at the Key Laboratory of Ion Beam Bioengineering (LIBB) of the Chinese Academy of Sciences (CAS) [18,19]. There are also two sites in France with microbeam facilities capable of irradiating individual animals. Proton microbeam irradiation of *C. elegans* embryos has been carried out at the Applications Interdisciplinaires des Faisceaux d’Ions en Région Aquitaine (AIFIRA) facility of the Centre d’Études Nucléaires de Bordeaux Gradignan (CENBG) at the University of Bordeaux [20,22,30]. The proton microbeam irradiation of *C. elegans* individuals has also been carried out at the Institut de Radioprotection et de Sûreté Nucléaire (IRSN), where irradiation facilities for proton and heavy-ion microbeam irradiation are maintained [21,31].

## 3. Current Status of Microbeam Irradiation of Live *C. elegans*

### 3.1. Previous Methods for C. elegans Immobilization

In *C. elegans* research, the role of each cell and/or cell–cell interaction has been explored by selective cell ablation with a laser microbeam [32,33,34,35,36,37]. In addition, because the body is transparent and organs can be easily observed, various transgenic nematodes have been produced and fluorescence observation of cell dynamics has been widely used.

*C. elegans* is a thread-like animal of approximately 1 mm in length that crawls with a head-swinging motion, moving forward and backward, and changing direction. Since it is essential to suppress this movement during laser irradiation of specific cells of live *C. elegans* and observation of cell dynamics, several methods of immobilization have been established.

**Figure 2:**
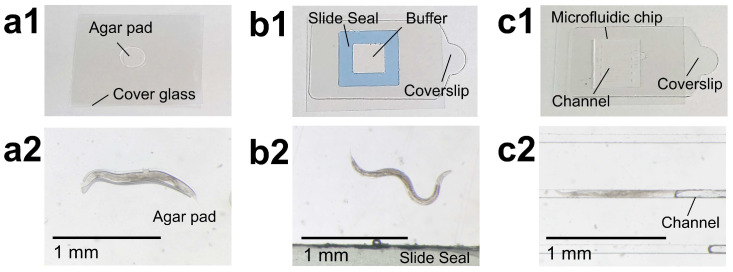
Schematic of the *C. elegans* immobilization methods. (**a1**) Agar pad. (**a2**) Adult *C. elegans* on an agar pad covered by a cover glass. (**b1**) Recess using a slide seal containing buffer solution. (**b2**) Adult *C. elegans* enclosed in the recess covered by a coverslip (cover film). (**c1**) Microfluidic chip named a Worm Sheet on a cover glass. (**c2**) Adult *C. elegans* enclosed in a straight microfluidic channel of a Worm Sheet. Bars represent 1 mm.

The most common method is retention on agarose gel pads [2–10% (*w*/*v*)] or agar pads [2–5% (*w*/*v*)]. A drop of agarose or agar solution is placed on a glass slide or cover glass, which is flattened and solidified, and the animals are placed on top and covered (Figure 2(a1,a2)). An anesthetic such as sodium azide or levamisole may be added to control movement more reliably. Alternatively, the animals can simply be held in place using anesthesia alone.

However, in studies of muscle movement and higher neural functions, it is undesirable for the anesthetic to be retained and inhibit cellular activity, even temporarily. To address this issue, a method for immobilizing *C. elegans* on agarose pads using polystyrene nanoparticles instead of anesthesia has been established [38,39,40]. A method using a non-ionic triblock copolymer, which changes from a liquid at 4 °C to a gel when heated to room temperature, has also been established [22]. The effects of liquids and gels attached to the *C. elegans* body and the long-term physical stimulation might be problematic in behavioral experiments investigating locomotion and/or stimulus responses immediately after irradiation, but it is suitable for developmental research and other applications.

In addition, especially for long-term live imaging observation, it is important to keep the animals straight and prevent small movements. To address this, a novel protocol has been proposed for the production of grooved agarose pads using a 12″ vinyl long-play (LP) record as an agar pad mold in which animals can be positioned and immobilized for live imaging [41]. Another method is to use a slide seal to create a recess several hundred microns deep to encapsulate *C. elegans*, along with the buffer solution [10] (see Figure 2(b1,b2)).

For heavy-ion and/or proton microbeam irradiation, the samples must be sufficiently thin to allow the beams to pass through them, and the use of agarose gel/agar pads is not appropriate. In addition, the continuous physical stimulation caused by the agarose gel/agar pad and the cover sandwiched between them is not negligible. To overcome this, a less stressful method of holding and handling the animals for microbeam irradiation is required.

### 3.2. PDMS Microfluidic Chips for Immobilizing Live C. elegans for Microbeam Irradiation

To suppress the movement of *C. elegans* individuals and precisely irradiate only the targeted area with the charged-particle microbeam, an on-chip immobilization method was developed, focusing on microfluidic devices/chips made of polydimethylsiloxane (PDMS) [17,42,43]. PDMS microfluidic chips have microfluidic channels ranging from a few microns to several tens-of-microns in width. The microfluidic channels are formed by transferring arbitrarily structured microfluidic channels onto the surface, and are biocompatible. In the case of a microfluidic device developed by a research group at Columbia University, *C. elegans* individuals are introduced into the device through liquid flow between an inlet and an outlet, and the size of each microfluidic channel guarantees that young adult animals are immobilized within minutes without the use of anesthesia. After site-specific irradiation with the microbeam, the animals can be released by reversing the flow direction in the clamp, and collected for analysis [17]. The straight microfluidic channels (ditch) can accommodate animals without affecting their physiological activity and only suppress their movement, allowing region-specific microbeam irradiation. Although this system is useful for *C. elegans* immobilization, if there is not enough space on the irradiation device, it cannot be installed. In addition, the device itself is thick, and charged particles cannot pass through it, making it difficult to employ it on an instrument that detects charged particles on the underside of the sample.

A QST-TIAQS research group has developed a simpler structure that does not have inlets and outlets for the introduction and removal of buffer solution, and a sheet-like microfluidic chip that is thinner than the range of carbon ions in water, although the concept of confining and retaining animals in a straight microfluidic channel is the same. PDMS is a highly self-adhesive material and can be easily sealed with a thin cover possessing a smooth surface, suppressing the movement of the accommodated *C. elegans* individuals. This enables the creation of a microfluidic chip with a simple structure consisting of only the chip and a cover film. Furthermore, the hydrophilic treatment of PDMS, which is inherently hydrophobic, has been shown to confer water-retention properties on the microfluidic chip, preventing dehydration of the *C. elegans* individuals and allowing them to be housed and maintained in a viable state for long periods of time. An example of the developed PDMS microfluidic chip “Worm Sheet” [44,45] is shown in Figure 2(c1,c2). At 15 mm long and 15 mm wide, the Worm Sheet has 20-to-25 straight microfluidic channels on its surface, which is almost the same size as the width of the adult *C. elegans* body (60 or 50 µm), allowing multiple animals to be trapped and retained simultaneously.

The Worm Sheet retains *C. elegans* by covering the microfluidic channels formed on its surface for *C. elegans* containment with a cover film. There are no particular restrictions on the cover film, as long as it is transparent and does not interfere with microscopic observation and the surface is smooth and adheres to the PDMS material of the microfluidic chip. The Worm Sheet’s thickness was set to 300 µm, and, including the top and bottom films, which are each approximately 100 µm thick, it is approximately 500 µm. This is sufficiently thinner than the range of various heavy-ion beams in water, to allow heavy ions to penetrate it. The number of carbon ions hitting the *C. elegans* enclosed in the microfluidic channel of the Worm Sheet can be detected and accurately counted by the transmission ion counter of the irradiation system located below the sample, allowing microbeam irradiation with precisely controlled doses [44]. While the range of carbon ions in water at TIARA is approximately 1.2 mm (^12^C^5+^, 220 MeV) [11] or 930 µm (^12^C^6+^, 190 MeV) [13], the range of protons in water is less than 150 μm. For proton irradiation, it is possible to use thin films with a thickness of 10 µm or less, such as a polypropylene film and Kapton film. While it is not possible to detect the number of protons hitting the *C. elegans* enclosed in the Worm Sheet in systems that detect protons passing through a sample at the bottom of the sample due to the thickness, it can be used in systems that detect protons at the top of the sample, or in systems that control the number of protons based on a pre-measured fluence rate.

Consideration was given to the type of buffer used for *C. elegans* immobilization, and also to cover films that effectively prevent acid deprivation during encapsulation, with the use of gelatin-containing buffers and highly oxygen-permeable polystyrene covers being advocated [45]. To assess the effects on *C. elegans*, the contraction and relaxation activities of body-wall muscle cells of this species enclosed in microfluidic channels were examined by calcium imaging and it was confirmed that the contraction and relaxation of the muscle cells were normal. In addition, whole-body locomotion of *C. elegans* was examined immediately after enclosing them in microfluidic channels for 1 h. It was confirmed that there were no significant adverse effects such as reduced locomotion [44].

### 3.3. Status of Microbeam Irradiation of C. elegans at Each Site

With advances in techniques for irradiating biological samples with narrowly focused heavy ions or protons, cell-to-cell irradiation has also been attempted. The main difference between individual animal irradiation and cell irradiation is that the target is constantly moving. To target specific miniscule areas for irradiation while maintaining a live state, it is necessary, apart from microbeam irradiation methods, to suppress only the movement of the *C. elegans* individuals without exposing them to additional stress. The heavy-ion or proton microbeam irradiation of live *C. elegans* reported to date is summarized in Table 1, focusing on the immobilization method and the use of anesthesia, and is also outlined below.

The first microbeam irradiation experiment of live *C. elegans* individuals at the JAEA- Takasaki (now QST-TIAQS) was reported in 2006 for carbon-ion irradiation targeting the gonad and tail [10]. In this experiment, a slide seal (a frame with adhesive seals on both sides) was pasted on a slide glass and filled with buffer solution containing an anesthetic (sodium azide). The *C. elegans* individuals were released into the buffer and a coverslip was placed on the slide seal (see Figure 2(b1)). The gonad and tail of the live *C. elegans* individuals were irradiated [10].

Subsequently, in 2009, proton irradiation of the tail was reported using a RARAF microbeam at Columbia University. In this case, the *C. elegans* individuals were enclosed in a custom-made microbeam dish with a micro coverslip, together with an anesthetic (sodium azide), to inhibit movement [16]. This experiment highlighted the problem of using anesthesia to suppress *C. elegans* movement. In light of this, in 2013, the research group developed a microfluidic device for *C. elegans* observation and irradiation without anesthesia, and firstly conducted microbeam irradiation of live *C. elegans* individuals without anesthesia [17]. Coincidentally, at about the same time, a research group at QST-TIAQS, independently of Columbia University, began research and development on the use of microfluidic chips to immobilize and irradiate live *C. elegans*.

In 2013 and 2016, the proton microbeam facility at CAS-LIBB was used for proton irradiation of the posterior pharyngeal bulb, tail, and rectal valve of *C. elegans*. In these cases, individuals were placed on 2% agarose gels with an anesthetic (mixture of ethanol and ethyl ether) to immobilize them [18,19].

As described above, in research targeting motor and higher neural functions, where immediate post-irradiation analysis is important, techniques are needed to irradiate target areas without anesthetic being retained and with reduced movement. Therefore, at QST-TIAQS, an original *C. elegans* immobilization method for microbeam irradiation was devised and at TIARA of QST-TIAQS, carbon-ion irradiation targeting the head, intestines, and tail using a collimated microbeam device was reported in 2017 [11]. This was the second example of microbeam irradiation of live *C. elegans* individuals without anesthesia and without affecting neural or muscle activity. To immobilize the *C. elegans* individuals, they were enclosed in straight microfluidic channels formed on a PDMS microfluidic chip. The hydrophobic PDMS microfluidic chip used in this case made it difficult to maintain *C. elegans* in a good condition over time because they became dehydrated by encapsulation in the microfluidic channel.

In 2018, to prevent dehydration of the *C. elegans* individuals due to long-term immobilization in microfluidic channels, the sheet-type PDMS microfluidic chip Worm Sheet with unique water-retention properties was developed as described above, which enables the immobilization of animals without anesthesia [44,45].

In 2019, proton irradiation of two-cell-stage embryos using the CPM at the AIFIRA facility of the CNRS/IN2P3 and the University of Bordeaux was reported. In this case, stretched polypropylene foils were used without anesthesia [20].

In 2020, carbon-ion irradiation targeting head neurons using QST-TIAQS’s focused microbeam device at TIARA was reported [12]. In this example, animals were held without anesthesia in the straight microfluidic channels of a proprietary retentive PDMS microfluidic chip and irradiated with targeting of a single neuron. In the same year, carbon-ion irradiation of the central nervous system using QST-TIAQS’s collimated microbeam system at TIARA was reported. Again, a new experimental analysis system was presented in which *C. elegans* individuals were experimented on without anesthesia in straight microfluidic channels of a water-retaining PDMS microfluidic chip and a series of experimental analyses were performed on-chip: fluorescence observation of muscle activity before irradiation, and targeted irradiation and fluorescence observation of muscle activity after irradiation [13]. In addition, carbon-ion irradiation of the anterior and posterior halves of the body using a similar method was reported in 2021 [14].

In 2023, the first proton microbeam irradiation of *C. elegans* individuals at the IRSN’s MIRCOM facility was reported. The *C. elegans* individuals were encapsulated in coverslips and polypropylene sheets containing 2% agarose gel, their movement was inhibited by an anesthetic (levamisole), and the irradiation was targeted at the head [21].

In the same year, proton irradiation targeting gonadal stem cells was reported at the AIFIRA facility. In this case, L1-stage *C. elegans* populations were placed in a specific mounting medium consisting of M9 medium supplemented with tetramisole hydrochloride and a non-ionic triblock copolymer, poloxamer 407. Poloxamer-407 changes from a liquid at 4 °C to a gel when heated to room temperature (~20 °C), allowing *C. elegans* to be immobilized during irradiation and recovered quickly and easily after irradiation [22].

In addition, in 2023, carbon-ion tissue shape-fill irradiation of the pharynx and gonad using QST-TIAQS’s focused microbeam device at TIARA was reported. In this case, nematodes were enclosed in straight microfluidic channels of a water-retentive PDMS microfluidic chip, together with a solution of anesthetic (sodium azide) at a very low concentration, to inhibit movement [15].

As described above, charged-particle microbeam irradiation of live *C. elegans* has so far been achieved in Japan, the USA, China, and at two sites in France. Researchers at Columbia University, QST-TIAQS and the University of Bordeaux have achieved microbeam irradiation of live *C. elegans* individuals without anesthesia, and the standardization of immobilization techniques will make this feasible on a global scale. In addition, the next step would be to implement an integrated vision using image processing and a stage control system that tracks and irradiates freely moving animals without holding them in place.

### 3.4. A Wide Range of Microbeam Biological Research Using C. elegans

The microbeam irradiation experiments of *C. elegans* individuals or embryos conducted at the five sites (excluding QST-iQMS) cover a wide range of topics, including development, reproductive function, nervous-system function, and motor function. The technological and experimental topics at each site are outlined below.

A research group at QST-TIAQS (formerly JAEA-Takasaki), using two types of heavy-ion microbeams at TIARA, reported the effects of carbon-ion microbeam irradiation on reproductive function in 2006 [10], in 2017 and 2020 they achieved irradiation of the central nervous system while maintaining neuromuscular activity [11,13], and in 2021 they showed the dose and irradiation site at which high-dose heavy-ion half-body irradiation has irreversible effects on motor function [14]. They have also made remarkable technological innovations, and in 2020 they developed a technology to irradiate a single neuron [12], while in 2023 they developed a technology to irradiate specific tissues of arbitrary shape, such as the pharynx and reproductive organs [15]. In addition, they are currently establishing a system to enable research on the effects of heavy-ion irradiation on all life functions of individual *C. elegans*.

A research group at Columbia University, using RARAF’s proton microbeam, first applied proton microbeam irradiation to a specific part of the tail of young *C. elegans* to study the stress response in 2009, with the goal of studying the radiation-induced bystander effects at the individual level [16]. In 2013, they developed a microfluidic device for microbeam irradiation of multiple live *C. elegans* one after the other, and indicated that the proton microbeam induced DNA damage in the wild type [17].

A research group at CAS-LIBB, using a proton microbeam facility, reported that proton microbeam irradiation of posterior pharynx bulbs and tails of *C. elegans* enhanced the level of germ cell apoptosis in bystander gonads, and posterior pharynx bulb irradiation also increased the level of DNA damage in bystander germ cells and genomic instability in the F_1_ progeny of irradiated animals in 2013 [18]. In 2016, they reported that proton microbeam irradiation of the posterior pharynx bulbs and rectal valves of *C. elegans* could significantly suppress the induction of vulva protrusion by subsequent gamma irradiation, suggesting a contribution of radiation-induced bystander effects to radio-adaptive responses in the context of the whole organism [19].

Meanwhile, a research group at the University of Bordeaux, using the AIFIRA facility, established both a methodology to manipulate and irradiate early-stage *C. elegans* embryos at a controlled dose using a charged-particle microbeam, and a system for evaluating radiation-induced damage during embryonic development [20]. In 2023, they focused on gonadal and vulval development, and designed an efficient strategy for proton microbeam-targeted irradiation of specific stem cell regions in multicellular *C. elegans* individuals, to analyze the impact of radiation on organ development [21].

Furthermore, a research group at IRSN, using an irradiation facility for proton microbeam irradiation, investigated unknown mechanisms underlying structural and functional changes in the brains of patients treated with protons. In 2023, they reported an analysis of the effects of proton irradiation in the central nervous system of *C. elegans* with a focus on mitochondrial function and reported that protons induced mitochondrial dysfunction [22].

As summarized above, the endpoints of the microbeam irradiation experiments with *C. elegans* include DNA and mitochondrial damage, developmental abnormalities, behavioral abnormalities, and recovery, which are used to evaluate the biological effects of ionizing radiation. In addition, there is no single topic of focus across the five research sites, but rather several topics unique to each center, and it is expected that research will evolve in different directions, such as through comparative experiments with heavy ions and protons.

## 4. Discussion

### 4.1. Standardizing and Popularizing the Microbeam Irradiation Technology

Life science research using heavy-ion and proton microbeams has not yet progressed beyond the realm of analyzing the effects of ionizing radiation at any research institute globally. However, the accumulation of findings from such ongoing research to deepen our understanding of the effects of microbeams on individual animal cells and tissues and their mechanisms of action will undoubtedly be useful in establishing valuable next-generation technologies for temporarily suppressing or increasing the activity of cells and tissues without destroying them.

Although it is not easy to achieve international standardization of irradiation techniques, due to the unique circumstances of individual facilities and equipment, it is possible to reduce the number of techniques and fields of expertise that each site must develop independently by promoting common protocols for experimental techniques, such as the *C. elegans* immobilization method for microbeam irradiation. For example, the research group at QST-TIAQS has also attempted to standardize and popularize its own technology around the world. The above-mentioned microfluidic chip for adult *C. elegans* has been made commercially available as a standardized product named “Worm Sheet” [44,45] from a collaborator, Biocosm Inc. (Amagasaki, Japan), and can be obtained from outside of Japan. It is also possible to request Worm Sheet with desired specifications, such as having microfluidic channels of a size suitable for eggs or larvae, and it is possible to customize the Worm Sheet for ease of use according to the content of the experiment and the circumstances of the experimental equipment.

For the microbeam technology developed at a particular institution to become a common resource universally used, it is necessary to have a mutual understanding of the details of the equipment at the world’s microbeam sites, the content of the experiments conducted so far, and the issues currently facing researchers in this field. To make this possible, it will be necessary to review each device, as well as to form and strengthen the activities of an international microbeam consortium that will serve as a forum for information exchange and discussion.

Standardization of technology also promotes the spread of microbeam technology to related fields. As well as the biofunctional analysis based on heavy-ion and proton irradiation described in this paper, experimental analysis systems have been developed for the irradiation of microorganisms such as *C. elegans*. For example, elementary technologies already established for UV microbeam irradiation to target specific cells/tissues [46], time-resolved X-ray observation for *C. elegans* analysis [47], and synchrotron X-ray microbeams in a radiotherapeutic application known as microbeam radiotherapy (MRT) [48] can be applied to heavy-ion/proton microbeam irradiation. In the case of irradiation of specific cells and tissues of live *C. elegans*, the immobilization method occupies an important position, along with carbon-ion/proton microbeam irradiation. In fact, the water-retentive PDMS microfluidic chip called Worm Sheet described in this paper, which was developed for heavy-ion microbeam irradiation, has been used as an immobilization tool for fluorescence imaging of cell morphology and dynamics [49] and as an immobilization device for measuring the temperature of *C. elegans* using quantum sensing [50].

### 4.2. Evaluation of Results of a Limited Number of Microbeam Experiments on Living Animals

Nearly 20 years have passed since the first report of microbeam irradiation of *C. elegans* individuals in 2006 [10], but fewer than 20 peer-reviewed papers on microbeam irradiation of *C. elegans* have been published, as shown in Table 1. The problem with microbeam irradiation experiments is that it is not always easy to repeat the experiment and obtain statistically reliable data. There are two main reasons for this: first, the experiment itself is extremely advanced and the throughput cannot be increased, and second, there are problems because the available beam time is extremely limited, so the number of experiments cannot be increased. This situation will not improve overnight. If the general standards for biological experiments are applied and evaluation is not performed unless statistically reliable data are available, the barriers to the dissemination of biological microbeam irradiation research in the developmental stage will be even higher.

As shown in Table 1, the number of samples (individuals irradiated) per trial and the number of trials (experimental replicates) vary. This is limited to experiments that have been accepted and published as peer-reviewed papers, but, in reality, there are probably many more data sets that have not been published due to lack of data. It would be valuable if, as part of international standardization, there were discussions about what level of data should be considered reliable for microbeam irradiation experiments.

## 5. Conclusions

This article outlines the current status of the charged-particle microbeam irradiation experiment with *C. elegans* at five sites around the world, focusing on the targeted microbeam irradiation to specific sites in live *C. elegans*. In 2013, a research group at Columbia University succeeded for the first time in irradiating *C. elegans* without anesthesia while maintaining nervous system and muscle activity, and technology for the charged-particle microbeam irradiation of *C. elegans* is evolving year by year. Making one’s own technology available to everyone should greatly contribute to expanding the scope of microbeam technology development and microbeam biology.

Microbeam irradiation makes it possible to deliver a single charged particle to a biological sample or to irradiate only specific tissues of an individual. Therefore, charged-particle microbeam irradiation with *C. elegans* will be increasingly important in studies elucidating the effects of extremely low doses of radiation, which is key for assessing the effects of space radiation [7] and radiation exposure from various accidents, as well as for studies elucidating the effects of irradiation on specific organs in cancer therapy [2].

To this end, it is vital to accelerate the technological development of live-animal microbeam irradiation through interdisciplinary and international technology exchange.

## Figures and Tables

**Table 1:** Overview of live *C. elegans* irradiation with microbeams.

Facility	Radiation	Beam Spot Diameter	Targeted Region	Number of Samples * /Trial	Number of Trials *	Immobilization Device or Medium	Anesthesia	Year Ref.
Collimated microbeam device of TIARA (JAEA)	Carbon ions	20 µm	Posterior gonad	12	1	Slide Seal and a coverslip	Sodium azide	2006 [10]
	Carbon ions	20 µm	Tail	12	1	Slide Seal and a coverslip	Sodium azide	
RARAF microbeam (Columbia Univ.)	Protons	0.5 µm	Tail	≥20	-	Customized microbeam dish with a micro coverslip	Sodium azide	2009 [16]
RARAF microbeam (Columbia Univ.)	Protons	5 µm	head/intestine	(16)	-	16-channel microfluidic device	-	2013 [17]
Proton microbeam facility (CAS-LIBB)	Protons	8.2 µm	Posterior pharynx bulb	≥20	3	2% of agarose gels	Mixture of ethanol and ethyl ether	2013 [18]
	Protons	8.2 µm	Tail	≥20	3	2% of agarose gels	Mixture of ethanol and ethyl ether	
Proton microbeam facility (CAS-LIBB)	Protons	8.7 µm	Posterior pharynx bulb	≥4	-	2% of agarose gels	Mixture of ethanol and ethyl ether	2016 [19]
	Protons	8.7 µm	Rectal valva	≥4	-	2% of agarose gels	Mixture of ethanol and ethyl ether	
Collimated microbeam device at TIARA (QST)	Carbon ions	20 µm (beam exit)	Head	5	7	PDMS microfluidic chip	-	2017 [11]
			Intestines	5	7	PDMS microfluidic chip	-	
			Tail	5	7	PDMS microfluidic chip	-	
CPM at the AIFIRA facility (CNRS/IN2P3 and The Univ. of Bordeaux)	Protons	1.5 µm	2-cell-stage embryo	9	-	Stretched polypropylene foils	-	2019 [20]
Focused microbeam device at TIARA (QST)	Carbon ions	8 µm	Head neuron	(5)	-	Wettable PDMS microfluidic chip	-	2020 [12]
Collimated microbeam device at TIARA (QST)	Carbon ions	20 µm (beam exit)	Central nervous system	6	5	Wettable PDMS microfluidic chip	-	2020 [13]
		60 µm (beam exit)	Central nervous system	6	5	Wettable PDMS microfluidic chip	-	
Collimated microbeam device at TIARA (QST)	Carbon ions	60 µm (beam exit)	Anterior half body	8	1	Wettable PDMS microfluidic chip	-	2021 [14]
		60 µm (beam exit)	Posterior half body	9	1	Wettable PDMS microfluidic chip	-	
MIRCOM facility (IRSN)	Protons	2.2 ± 0.3 µm	Head	67–81	2	Coverslips and polypropylene sheet containing 2% agarose gel	Levamisole	2023 [21]
CPM at the AIFIRA facility (CNRS/IN2P3 and The Univ. of Bordeaux)	Protons	4 µm	Gonad stem cells	301–514	≥3	Afresh agar pad (3% (*w*/*v*)) and Poloxamer-407	-	2023 [22]
Focused microbeam device at TIARA (QST)	Carbon ions	-	Pharynx	(1)	-	Wettable PDMS microfluidic chip	Sodium azide	2023 [15]
		-	Gonad	(1)	-	Wettable PDMS microfluidic chip	Sodium azide	

* When there were two or more microbeam experiments (including different conditions), the basic experiment (the one with the larger number) is listed. If there was no clear description and the value was estimated based on the method description, among other factors, the value is given in parentheses.
